# Identification and Biological Evaluation of CK2 Allosteric Fragments through Structure-Based Virtual Screening

**DOI:** 10.3390/molecules25010237

**Published:** 2020-01-06

**Authors:** Chunqiong Li, Xuewen Zhang, Na Zhang, Yue Zhou, Guohui Sun, Lijiao Zhao, Rugang Zhong

**Affiliations:** 1 Beijing Key Laboratory of Environmental & Viral Oncology, College of Life Science and Bioengineering, Beijing University of Technology, Beijing 100124, China; chunqiong.li@emails.bjut.edu.cn (C.L.); zhangxuewen@emails.bjut.edu.cn (X.Z.); sunguohui@bjut.edu.cn (G.S.); zhaolijiao@bjut.edu.cn (L.Z.); lifesci@bjut.edu.cn (R.Z.); 2 State Key Laboratory of Bioactive Substances and Functions of Natural Medicines, Institute of Materia Medica, Chinese Academy of Medical Sciences & Peking Union Medical College, Beijing 100050, China; zhouyue@imm.ac.cn

**Keywords:** protein kinase CK2, allosteric fragments, αD pocket, virtual screening, anti-cancer hits

## Abstract

Casein kinase II (CK2) is considered as an attractive cancer therapeutic target, and recent efforts have been made to develop its ATP-competitive inhibitors. However, achieving selectivity with respect to related kinases remains challenging due to the highly conserved ATP-binding pocket of kinases. Allosteric inhibitors, by targeting the much more diversified allosteric site relative to the highly conserved ATP-binding pocket, might be a promising strategy with the enhanced selectivity and reduced toxicity than ATP-competitive inhibitors. The previous studies have highlighted the traditional serendipitousity of discovering allosteric inhibitors owing to the complicate allosteric modulation. In this current study, we identified the novel allosteric inhibitors of CK2α by combing structure-based virtual screening and biological evaluation methods. The structure-based pharmacophore model was built based on the crystal structure of CK2α-compound 15 complex. The ChemBridge fragment library was searched by evaluating the fit values of these molecules with the optimized pharmacophore model, as well as the binding affinity of the CK2α-ligand complexes predicted by Alloscore web server. Six hits forming the holistic interaction mechanism with the αD pocket were retained after pharmacophore- and Alloscore-based screening for biological test. Compound **3** was found to be the most potent non-ATP competitive CK2α inhibitor (IC_50_ = 13.0 μM) with the anti-proliferative activity on A549 cancer cells (IC_50_ = 23.1 μM). Our results provide new clues for further development of CK2 allosteric inhibitors as anti-cancer hits.

## 1. Introduction

Protein kinase CK2 is involved in multiple physiological processes by phosphorylation of many substrates as the cell survival promoter and apoptosis suppressor [1,2,3]. This kinase is considered to be the promising cancer therapeutic target, and many efforts have been made on the discovery of CK2 inhibitors for the cancer therapy.

**Figure 1:**
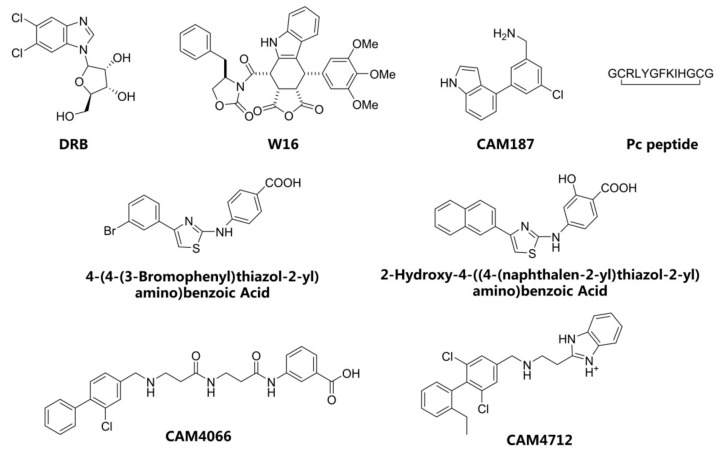
The structures of reported CK2 inhibitors binding to Non-ATP binding pocket.

The conventional strategy for CK2 inhibition focused on the discovery of orthosteric molecules that bind into the conserved ATP-binding site [4,5,6]. However, most of the known ATP-competitive inhibitors have been impeded to be the drug candidates due to the off-target and low selectivity [7,8,9,10]. Recently, the allosteric inhibitors targeting the sites out of the catalytic pocket are becoming an alternative strategy to develop the efficient and safe therapeutic agents [11,12]. For example, the subtype selective PDE4D allosteric inhibitor, BPN14770 exhibited the reduced vascular toxicity over earlier PDE4 inhibitors that lacked the subtype selectivity [13], and compound 8t as the allosteric inhibitor of phosphoglycerate mutase 1 was demonstrated to delay tumor growth in H1299 xenograft model without the obvious toxicity [14]. Identification of allosteric pockets is the basis for the design of allosteric inhibitors. Six allosteric sites of CK2 have been predicted by Jiang et al. using the combination of bioinformatics and biochemistry methods [15]. To date, three kinds of allosteric inhibitors were reported (show in Figure 1). CK2 α/β interface (Site 6 including Tyr 39, Val67, Val112 and Val101) has been confirmed to be occupied by the DRB, W16, CAM187 and cyclic peptide Pc, which antagonize the assembly of CK2 holoenzyme complex [16]. The αD pocket named site 3 was demonstrated as the allosteric pocket to accommodate the biaryl rings of CAM4712 analogues [17,18,19]. Recently, Bestgen et al. discovered aminothiazole derivatives as the allosteric modulators of CK2 by targeting the interface between the αC helix and glycine-rich loop [20,21]. However, most allosteric modulators were discovered by high-throughput screening experiment, which is time-consuming and serendipitous to some extent due to the lack of the comprehensive understanding about the allosteric sites and mechanisms [22]. With the development of computational allosteric prediction methods including the structure-based virtual screening and protein-modulator interactions assessment, an integrated computational and experimental approach provide a robust tool to aid in the allosteric inhibitors discovery [23,24,25].

In this study, we aimed to explore the discovery of novel allosteric inhibitors targeting the αD pocket of CK2α using structure-based virtual screening method. Specifically, structure-based pharmacophore model and Alloscore were used to screen ChemBridge fragment library, and then the screened hit candidates were subjected to an in vitro CK2 inhibition assay, as well as the anti-proliferative effects against cancer cells with the potential to serve as lead compounds for anti-cancer treatments.

## 2. Results and Discussion

### 2.1. Structure-Based Pharmacophore Modeling

**Figure 2:**
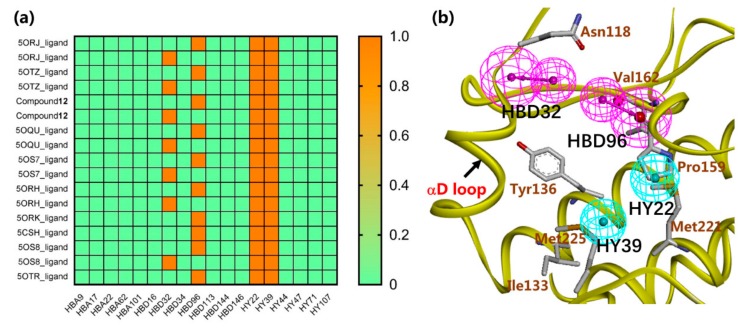
The four pharmacophoric features (**a**) HBD32, HBD96, HY22 and HY39 identified by the active compounds; and (**b**) mapped with the key residues of CK2αD pocket.

The structure-based pharmacophore model with 18 features was built based on the crystal structure of CK2α in complexed with compound 15 (compound number used in reference [19], PDB code: 5OTZ) using the “Interaction Generation” protocol in Discovery Studio 4.0. In order to optimize the pharmacophore model, ten active compounds reported in the literature were employed to hook the key pharmacophoric features as the positive set, and eight inactive compounds were used as the negative one [18,19] (Figure S1a). Two hydrophobic features (HY22 and HY39) were recognized by all the ten active compounds, and two H-bond (hydrogen-bond) donor features (HBD32 and HBD96) were hooked by eight out of ten active compounds (Figure 2a). Figure S1b showed the optimized pharmcophore model mapped with the ten active compounds. Obviously, the amino group perfectly mapped onto the two H-bond donor features, and the alkylbenzene ring rightly occupied the two hydrophobic features. Furthermore, the four features also corresponded to the key residues of αD pocket. Specifically, as shown in Figure 2b, two H-bond donor features matched with the backbone oxygen of Val162 or Pro159 and Asn118, and two hydrophobic features mapped on the hydrophobic pocket consisting of Ile133, Tyr136, Met221, and Met225.

### 2.2. Virtual Screening

**Figure 3:**
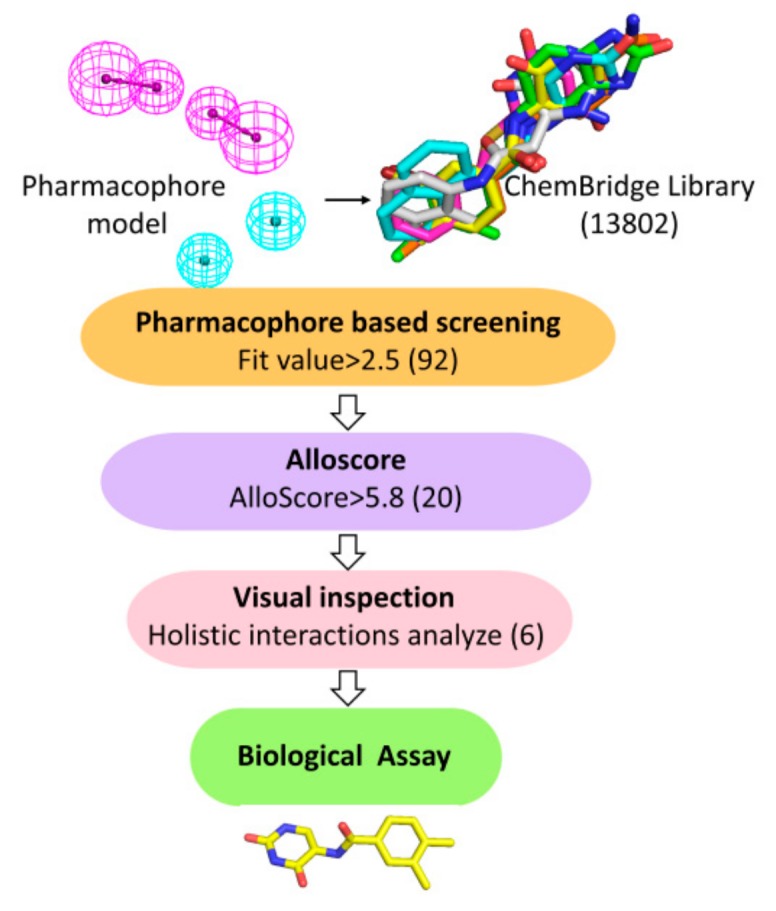
Virtual screening procedures.

Virtual screening was carried out following the workflow as indicated in Figure 3. The well-validated pharmacophore model was employed as a query for the retrieval of potent hits from ChemBridge Fragment Library (13802 compounds), and only 92 compounds were retained with the Fit values > 2.5.

Then the binding affinities of 92 molecules with CK2α were further evaluated by the web server Alloscore. As depicted in Figure S2, the AlloScore of active compounds were higher than 5.8, while six inactive compounds were lower than 5.8. Consequently, there is a good correlation between the AlloScore and the experimental Kd values among all the compounds, which validates the reliability and adaptability of the Alloscore scoring function as the screening filter. Based on the predicted AlloScore values of 92 compounds, 20 compounds that scored above any of the active compounds (5.8) were retrieved as the potential hits (Figure S3). By checking the holistic interactions and the diversity of chemical scaffolds of these fragments, six hits meeting the following requirements were retained for biological test: 1) compounds that form H-bonds with the Asn118, Pro159 or Val162, 2) compound could bind in the αD hydrophobic pocket consisted of Phe121, Tyr125, Ile133, Tyr136, Met221, and Met225, and 3) if compounds possess the similar scaffolds, some of them with the unreported pharmacophoric groups of αD pocket will be selected.

**Figure 4:**
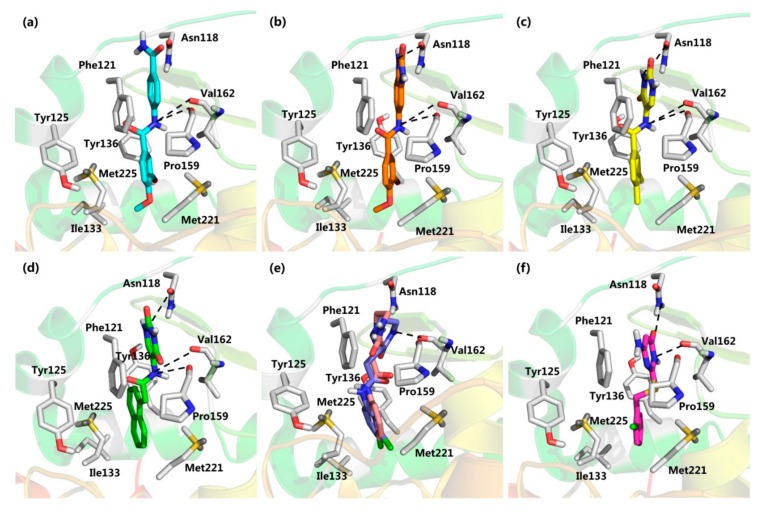
Docking poses of compound (**a**) **1** (cyan); (**b**) **2** (orange); (**c**) **3** (yellow); (**d**) **4** (green); (**e**) **5S** (purple) and R (pink); (**f**) **6** (magenta) in the αD site of CK2α.

Docking results presented the interaction modes of six hits with CK2α (Figure 4). Clearly, all the six hits located in the αD site, especially the ring B were entrapped by the hydrophobic pocket consisting of Phe121, Tyr125, Ile133, Tyr136, Met221 and Met225. The ring A and the linker –NH- of all the compounds formed polar interactions with the backbone atoms of Pro159 and Val162, which are also involved in the formation of H-bond with the linker NH of the CAM4066 analogues [18]. These compounds were bought from ChemBridge Corporation to test their inhibitory effect against CK2 and cancer cell proliferation.

### 2.3. CK2 Kinase Assay

**Figure 5:**
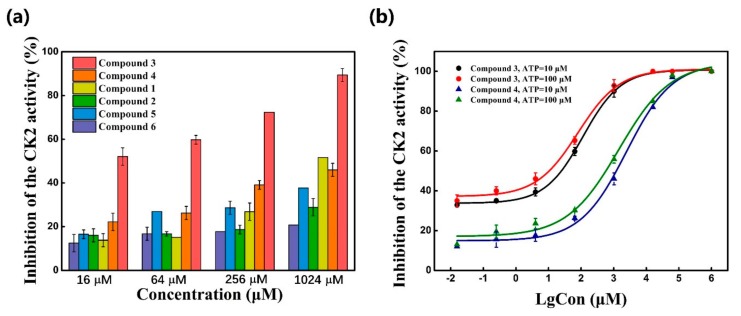
(**a**) Inhibitory activity of six compounds against CK2 at four concentrations; (**b**) Dose-dependent inhibitory effects of compound **3** and **4** against CK2 in the presence of 10 μM and 100 μM ATP.

In vitro CK2 kinase inhibition assay were carried out for the six compounds using ADP-Glo^TM^ kinase assay. Firstly, the six hits at four concentrations of 16 μM, 64 μM and 256 μM and 1024 μM were tested to investigate their rough inhibition potency against CK2α. As indicated in Table 1 and Figure 5a, these compounds presented the inhibitory effect on CK2α activity to different extent. At the concentration of 16 μM, compound **3** inhibited nearly 50% of CK2α activity. However, at the same concentration, other compounds had only 20% inhibitory activity. Furthermore, except compound **2** and **6**, the inhibition rates of these compounds were about 70% and 30% at 256 μM, respectively, with the dose-dependent manner.

Next, compound **3** with more than 50% CK2α inhibition at the concentration of 64 μM was put into the elaborate concentration-response studies, indicating the most potent inhibition of compound **3** on CK2α (IC_50_ = 13.0 μM). In contrast, compound **4**, although sharing the common 2,4(1H,3H)-pyrimidinedione (Uracil) group with compound **3**, was to be up to IC_50_ of 1024 μM. However, compound **5** (racemate) and **6** exhibited lower inhibition rates, below 50% at the concentration of 1024 μM. Following our results showing a potent inhibitory activity of compound **3**, we further investigated whether the inhibitory activity of compound **3** could be affected in the presence of 10 μM and 100 μM ATP. The similar IC_50_ values of 13.0 μM and 10.2 μM indicated that inhibitory activity of compound **3** was not influenced by 100 μM ATP (Figure 5b), which confirmed that this compound was a novel non-ATP competitive inhibitor of CK2.

**Figure 6:**
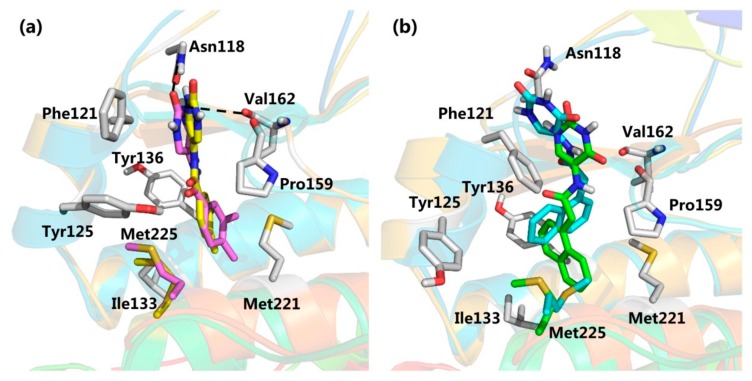
Superimposition of the docked conformation and the average structure from MD simulation: (**a**) compound **3** (yellow and pink, respectively); (**b**) compound **4** (green and cyan, respectively).

It is very interesting to explore the structure mechanisms for the different inhibitory activity of compound **3** and **4**. As indicated in Figure 6a, the pose of compound **3** in the MD simulation is extremely similar to that determined from the docking analysis. The skeleton of this compound fitted well into the αD pocket. The ring A formed the polar interactions with Asn118 and Val162, and the ring B was embedded into a hydrophobic cavity consisting of residues Ile133, Tyr136, Met221 and Met225. However, compound **4** could not bind into the αD pocket as compound **3** did, especially the ring B rotated out of the hydrophobic cavity (shown in Figure 6b). Maybe due to the steric clash between the larger size of naphthalene group (ring B) and the side chain of residue Met225, as well as the more flexibility of acetamide than amide, the naphthalene substituent tended to shift out of the hydrophobic pocket which accommodated the 3,4-dimethylphenyl (ring B) substituent of compound **3**. And also the flexible acetamide linker (compound **4**) may be unable to couple the ring A and B binding into the appropriate site as the rigid amide (compound **3**) could, which is consistent with the rigid biaryl groups as the αD site fragments [17].

### 2.4. Cell Proliferation Assay

**Figure 7:**
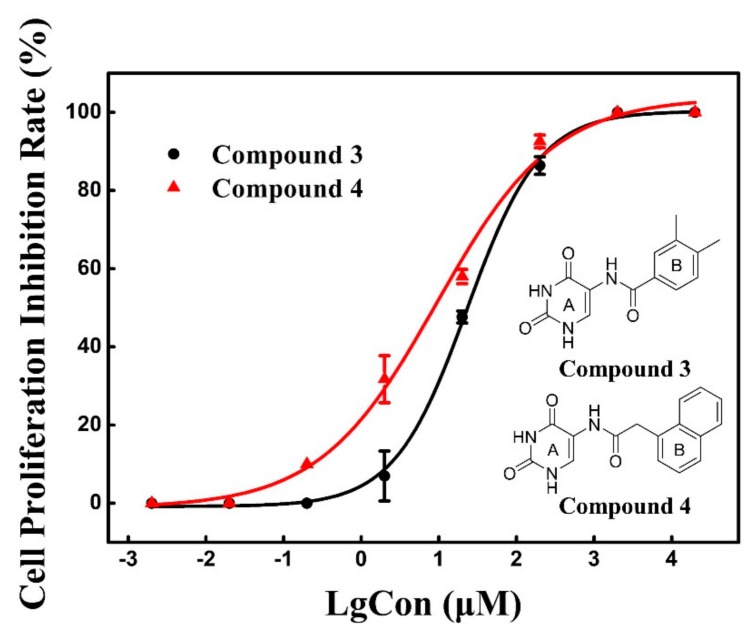
Dose response curve for the inhibition of A549 cell proliferation by compound **3** and **4**.

With the aim of investigating the anti-cancer cell proliferative activity of the CK2 inhibitors, compound **3** and **4** was tested against human cancer cell lines based on Cell Counting Kit-8 (CCK-8) assay. As shown in Figure 7, these two compounds exhibited a dose dependent response toward cell proliferation of A549 with the similar inhibitory activity of compound **3** (IC_50_ = 23.1 μM) and **4** (IC_50_ = 8.8 μM). We presumed that the functional uracil group of two compounds, which is also presented in the anti-cancer hits 5-fluorouracil [26], is essential and indispensable for anti-cancer activity.

However, two compounds generated the weak inhibitory effects on the proliferation of HeLa cells (IC_50_ > 100 μM) Hence, both of two compounds could be the potential drug candidates for the lung cancer although compound **4** was not regarded as the potent CK2 inhibitors.

## 3. Materials and Methods

Structure-based pharmacophore model was generated based on the αD cavity of the co-crystal structure of CK2α with compound 15 (compound number used in reference [19], PDB code: 5OTZ) by using the “Interaction Generation” protocol in Discovery Studio 4.0. And the 18 pharmacophoric features were identified according to the clustering analysis of the key residues. To get the optimized pharmacophore models, a set of ten active and eight inactive molecules were used to identify the key pharmacophoric features, and the features hooked by more than seven active compounds were selected.

The ChemBridge Fragment Library of 13802 commercially available compounds was used to perform structure-based screening with the workflow shown in Figure 3. Firstly, the well validated pharmacophore model was used as a 3D query to the potential hits, and only those fragments (92) with the fit value over 2.5 were further evaluated their binding affinity with CK2 using Alloscore web server [27]. Alloscore is a web server that predicts the binding affinities of allosteric ligand–protein interactions. This method exhibits prominent performance in describing allosteric binding and could be useful in allosteric virtual screening and the structural optimization of allosteric agonists/antagonists. The known ten active and eight inactive compounds [18,19] were predicted to explore the correlation between the AlloScore and experimental values. Secondly, in order to get the pre-docked structures of CK2-inhibitor complexes, GOLD 4.0 [28] software was used to dock the 92 fragments into CK2 αD pocket. Based on the binding affinities (AlloScore) of 92 retrieved hits, 20 hits scored above the active compounds were retained. Finally, taking the diversity of chemical scaffold into consideration, the top six hits that formed the holistic interactions with key residues were bought from ChemBridge Corporation for the following biological test. Molecular dynamics simulations were carried out for 30 ns using the Amber 14 package with the ff14SB force-field [29].

The ADP-Glo^TM^ Kinase Assay (Promega, Madison, WI, USA) was used to test the CK2 inhibition effects of these compounds [30,31]. As indicated by the relevant studies [32], competitive and noncompetitive inhibitors show the increased and similar IC_50_ values with increasing concentrations of ATP, respectively. Therefore, we were able to distinguish between ATP competitive and noncompetitive inhibitors of CK2 by exploring whether the IC_50_ value of inhibitors will change or not at 10 and 100 μM ATP. The kinase reaction was performed in 25 μL mixture containing 10 ng casein kinase 2α, 0.1 µg/μL casein protein, 10 µM or 100 µM ATP, 1× reaction buffer and serially diluted fragments (or DMSO for control). After 60 min incubation at room temperature, 25 μL of ADP-Glo^TM^ Reagent was added to terminate the kinase reaction and deplete the remaining ATP. Then 50 μL Kinase Detection Reagent was added to convert ADP to ATP within 30 min, and allow the newly synthesized ATP to produce a luminescence signal using luciferase/luciferin reaction, which was recorded by the luminescence panel of Microplate Reader (Enspire2300-001A, Perkin Elmer, Waltham, MA, USA).The luminescent signal is proportional to the ADP concentration produced and correlated with the kinase activity.

Cell Counting Kit-8 (CCK-8 Dojindo Laboratories, Kyushu, Japan) assays was used to assess the cytotoxic activities of compound **3** and **4**. A549 and HeLa cancer cells [33,34] were seeded in 96-well flat-bottom culture plate at 2 × 10^4^ cells/well and then cultured in an incubator of 5% CO_2_ and 37 °C for 24 h. After cells adherence, the cells were treated with targeted compounds at various concentrations for 48 h. Blank wells (containing culture medium only) and negative wells (untreated cells) served as controls. Following, 10 μL CCK-8 solutions was add to each well and incubated for 2 h at 37 °C, the absorbance was measured at 450 nm by using a microplate reader (Enspire 2300-001A, Perkin Elmer, Waltham, MA, USA).

## 4. Conclusions

In this study, compound **3**, with a novel scaffold, has been identified as a potent allosteric CK2 inhibitor by structure-based virtual screening of ChemBridge fragment library. The pharmacophore model including two H-bond donor and two hydrophobic features were used to screen the library with the filtration of fit values > 2.5. Then, considering the holistic recognition mechanism and binding affinity of fragments with CK2, docking studies and the Alloscore web server were used to further refine the screening result. Compound **3** was proved to be the potent CK2 inhibitors with an IC_50_ value of 13.0 μM tested by in vitro CK2 inhibition assay, and also exhibited the anti-proliferative effects against cancer cells (IC_50_ = 23.1 μM). These encouraging results provide the novel lead fragments for CK2 inhibitors, and also prompt the application of the structure-based discovery of anti-cancer allosteric drugs.

## Figures and Tables

**Table 1:** Structure, AlloScore and % inhibition of kinase activity of compounds.

Compound	2D Structure	AlloScore	%Inhibition at 16 μM	%Inhibition at 256 μM
**1**	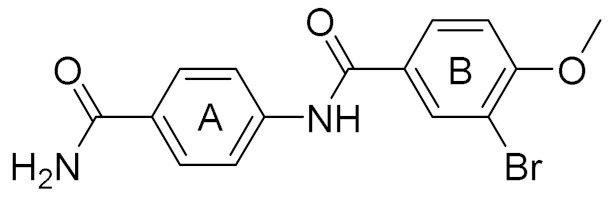	6.38	14	27
**2**	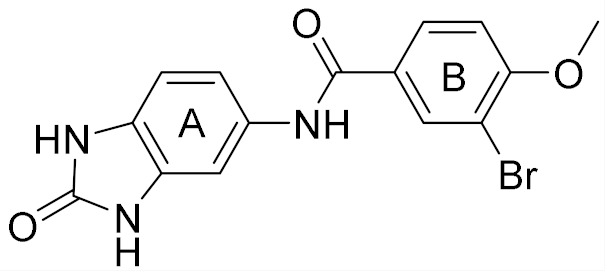	6.38	16	19
**3**	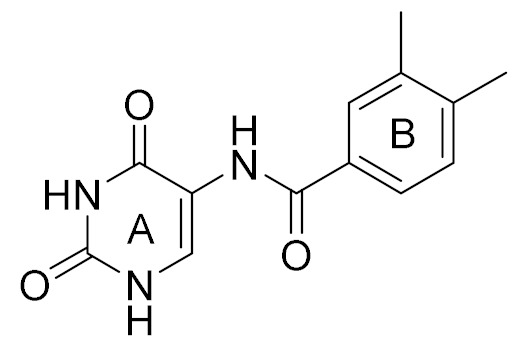	6.07	52	72
**4**	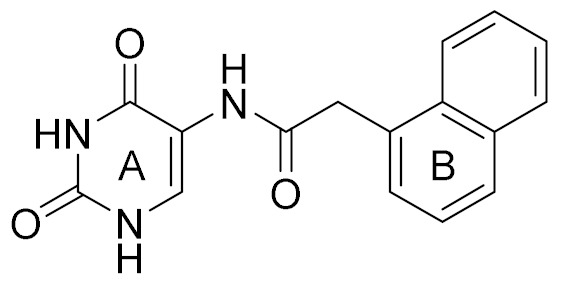	6.00	22	40
**5**	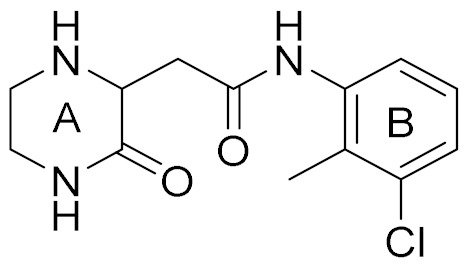	5.89 (S) 5.80 (R)	16	29
**6**	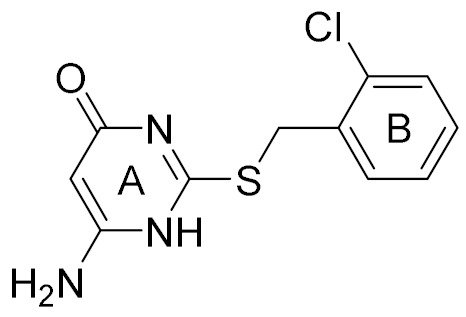	5.81	12	18

## Supplementary Materials

The following are available online, Figure S1: (a) Structures of ten active compounds and eight inactive compounds; (b) Superimposition of four pharmacophoric features on the ten active compounds. Figure S2: Correlation of AlloScore and the experimental Kd values. Figure S3: Structures of the selected 20 compounds with the AlloScore higher than 5.8.
