# Supplementary material for: Identification and Biological Evaluation of CK2 Allosteric Fragments through Structure-Based Virtual Screening
Source: Molecules. 2020 Jan 6;25(1):237. doi: 10.3390/molecules25010237 (PMC6983002; doi:10.3390/molecules25010237)
Supplement: Supplementary file 1 [file molecules-25-00237-s001.pdf]

Supplementary Materials

Identification and biological evaluation of CK2 allosteric fragments through structure-based virtual screening

Chunqiong Li[1], Xuewen Zhang[1], Na Zhang[1]*, Yue Zhou[2],Guohui Sun[1],Lijiao Zhao[1], and Rugang Zhong[1]

[1] Beijing Key Laboratory of Environmental & Viral Oncology, College of Life Science and Bioengineering, Beijing University of Technology, Beijing 100124, China; chunqiong.li@emails.bjut.edu.cn (C.L.); zhangxuewen@emails.bjut.edu.cn (X.Z.); nanatonglei@bjut.edu.cn (N.Z.);sunguohui@bjut.edu.cn (G.S.); zhaolijiao@bjut.edu.cn (L.Z.); lifesci@bjut.edu.cn (R.Z.)

[2] State Key Laboratory of Bioactive Substances and Functions of Natural Medicines, Institute of Materia Medica, Chinese Academy of Medical Sciences & Peking Union Medical College, Beijing 100050, PR China; zhouyue@imm.ac.cn (Y.Z.)

Corresponding author: Na Zhang

Tel.: +86-10-67392001 (N.Z.)

E-mail address: nanatonglei@bjut.edu.cn (N.Z.)

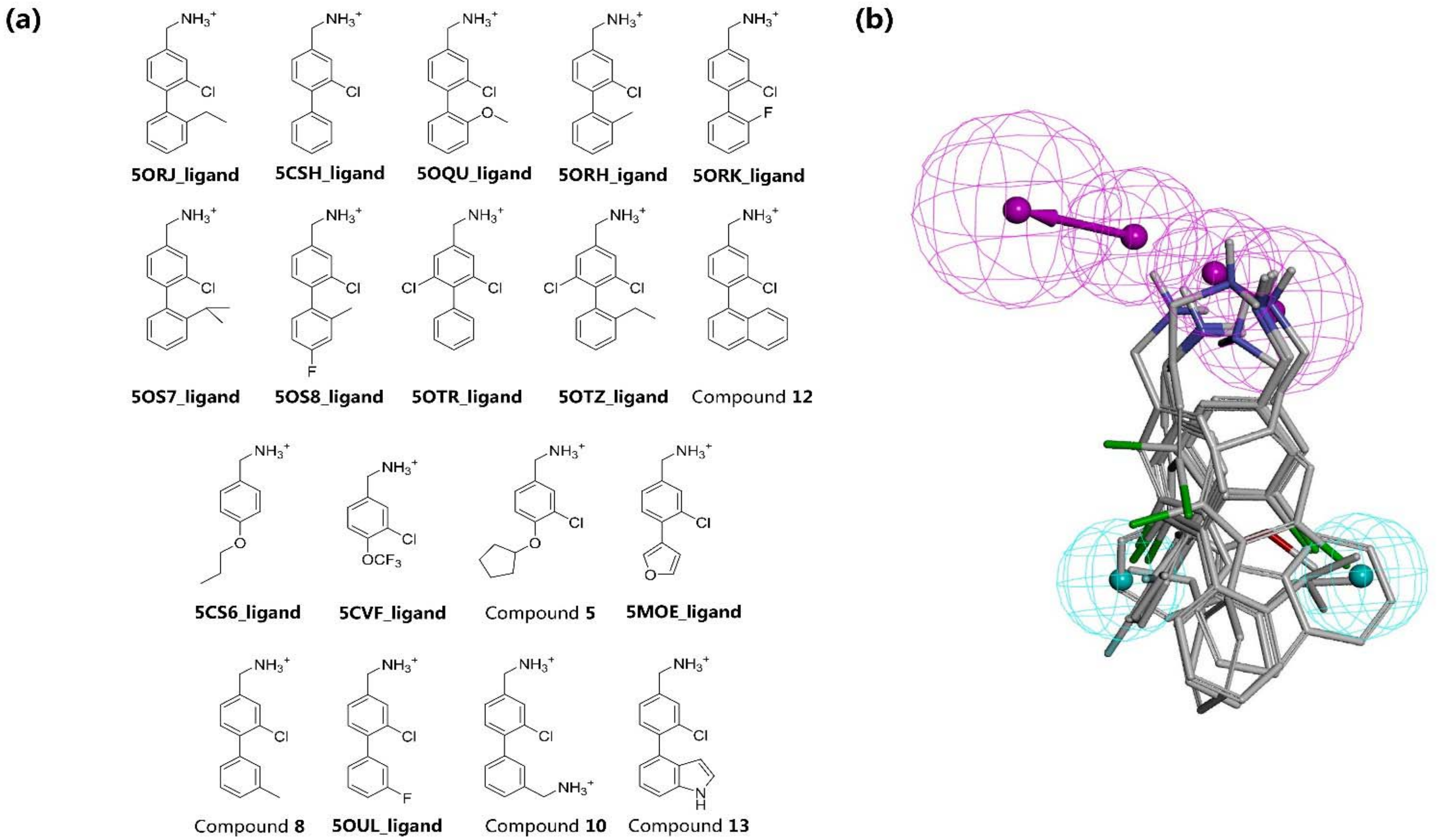

Figure S1. (a) Structures of ten active compounds and eight inactive compounds;

(b) Superimposition of four pharmacophoric features on the ten active compounds.

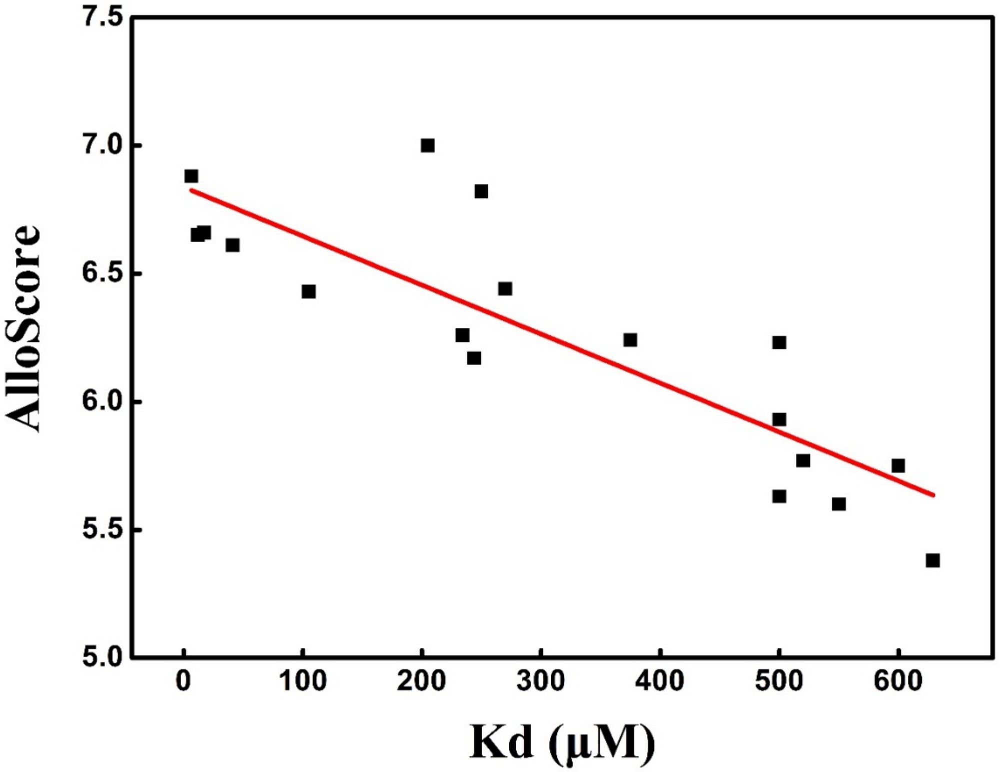

Figure S2. Correlation of AlloScore and experimental Kd values

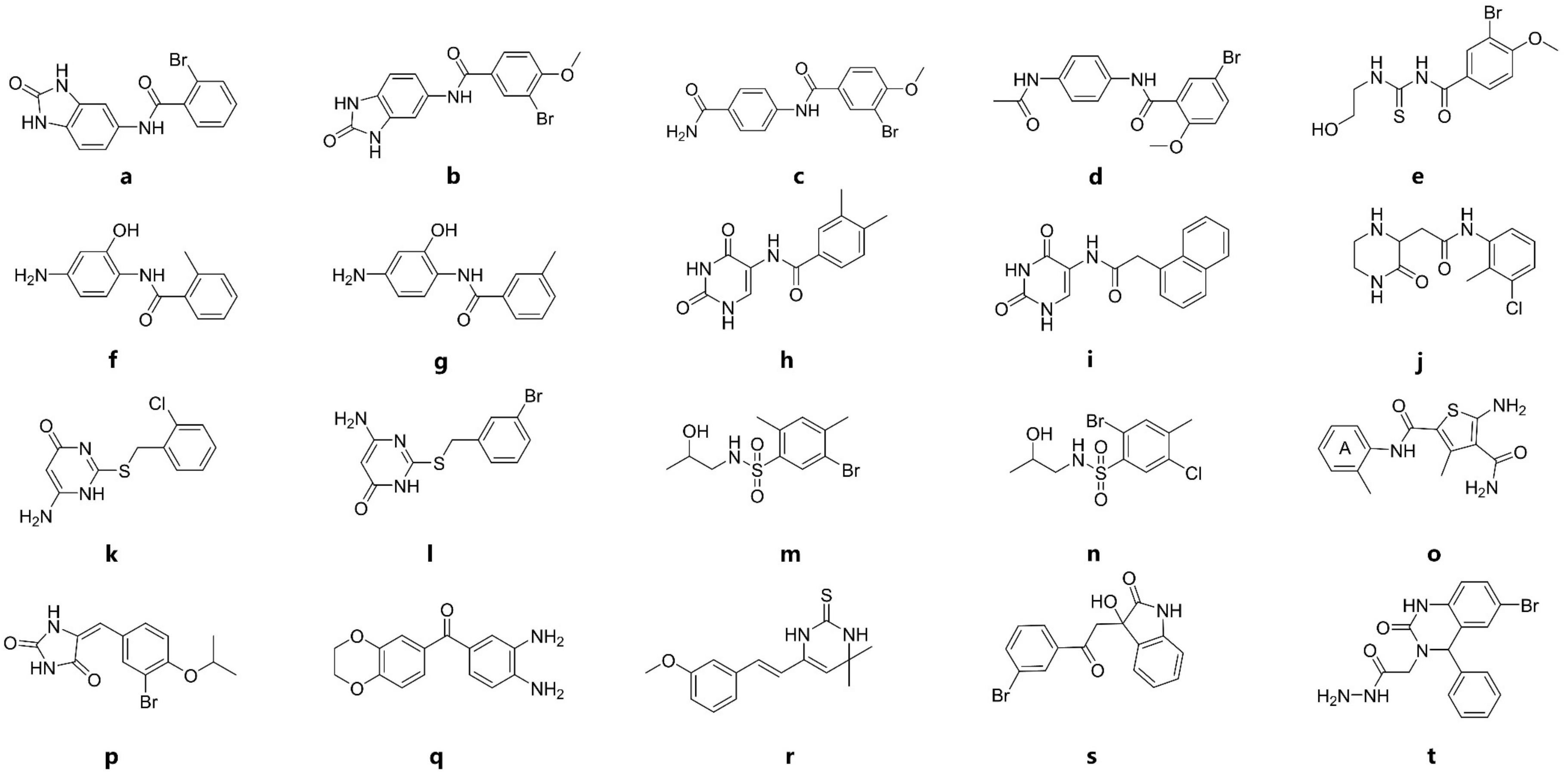

Figure S3. Structures of the selected 20 compounds with the AlloScore higher than 5.8.
